# Treasure of the Past: II: The Atomic Weight of Bromine

**DOI:** 10.6028/jres.105.038

**Published:** 2000-06-01

**Authors:** H. C. P. Weber

## INTRODUCTION

A considerable amount of work has been done in order to determine the atomic weight of bromine, and the oft repeated comparison of the atomic weights of silver and bromine makes it seem that this ratio is known with considerable accuracy. The value accepted for bromine, however, rests almost entirely upon that of silver, and it is of interest and importance to obtain a ratio between it and some other element. For chlorine a number of determinations of the ratio of hydrogen to chlorine in hydrochloric acid have been made, both by purely physical and by chemical methods. For bromine similar comparisons have not been made. Since the determination of the ratio chlorine : hydrogen was carried out with reasonable ease, it seemed probable that the method might be advantageously applied for the purpose of determining the ratio between hydrogen and bromine.

The method which was employed by Noyes and Weber^1^ was found to give good results in this case. The initial difficulties to be overcome were somewhat greater, which was rather unexpected. They were largely due to the physical properties of hydrobromic acid gas and were eliminated after the method had been studied for some time and slight alterations in the method of manipulation had been introduced.

It is not necessary to go into a discussion of previous work on bromine here. The entire subject matter is to be found either in Clarke’s “A Recalculation of the Atomic Weights”^2^ or in Abegg’s Handbuch, in the chapter on “Fundamental atomic weights.”^3^

The values which we have for bromine have been determined from the following ratios. The values here given are as given by Clarke, with one or two exceptions.

KBrO_3_ : KBr (Marignac), 80.291.
AgBrO_3_ : AgBr (Stas), 80.004.
Ag : Br (Marignac; Stas; Huntington; Richards; Richards, Collins, and Heimrod; Scott; and Baxter). In addition to these a considerable number of determinations have been made incidental to other work. These all taken together give a mean of 79.918.
AgCl : AgBr (Dumas, Baxter), 79.919.
Ag : KBr (Marignac; Stas; Dean; Richards and Mueller), 79.901.
AgBr : KBr (Richards and Mueller) (K=39.10), 79.903.
Ag : NH_4_Br (Scott, Stas), 79.935.
Ag : NaBr (Stas) (if Na=23), 79.961.

These values are calculated on the basis silver=107.88. From the determination of the ratio Na : Cl and Na : Br by Goldbaum,^4^ the value Br becomes 79.927 if sodium is taken as 23.00 and chlorine as 35.462. The value calculated by Clarke from all these determinations is 79.9197.

## PREPARATION AND PURIFICATION OF MATERIALS

### Hydrogen

The hydrogen used for the determinations was prepared by the electrolysis of a concentrated solution of barium hydroxide. The form of apparatus used has been described before,^5^ and was similar to the one used in the work on chlorine. The water used in the generator was “conductivity water.” The barium hydroxide was prepared by recrystallizing the purest commercial compound several times oftener than was necessary to free it of chlorine.^6^

The hydrogen generated in the electrolytic cell passed first through a heated hard glass tube, 40 cm long, filled with platinized quartz. Then it passed through a 30-cm length of fused potassium hydroxide, and then through three tubes, each about 30 cm long, containing resublimed phosphorus pentoxide. The apparatus was of glass throughout, connections between hard and soft glass being made by means of ground joints sealed together by means of a hard cement. When not actually in use the apparatus was sealed up.

### Bromine and hydrobromic acid

In preparing the materials it was desired to limit the reagents used in the process of purification to such as were strictly of the same degree of purity as the material being treated and further to employ such transformations only as would introduce no elements not desired in the final product. While the logic of this plan is obvious, it is not, or can not, always be followed in work of this character. It could not be followed strictly in this work. On the whole the transformations employed in the course of purification of the materials were largely physical. The reagents used in the preparation of the final material were limited as closely as possible to hydrogen, platinum, bromine, potassium salts and water. Aside from these reagents, formic acid was used for the reduction of the bromplatinate to platinum black, and oxalic acid in the form of potassium oxalate. In the case of potassium oxalate two fusions intervened before the final product, potassium bromide, was obtained.

The purification of the bromine and the hydrobromic acid may be considered as one case. The original material was an especially pure sample of bromine.^7^

Thirty-two pounds of this bromine were taken at the beginning of the work. This was distilled three times from a solution of potassium bromide (made from a portion of the same bromine and pure potassium oxalate). It was then cooled until all but a small portion had crystallized. The liquid was rejected. The crystals were allowed to melt gradually and the liquefied bromine was poured off from time to time until half of the bromine had liquefied. The further treatment of this liquefied portion will not be discussed. Although it was treated further in exactly the same way as the crystals from which it had been removed, it did not give so pure a product and was used only in the purification of the platinum. The solid portion melted at −7° C. It was again crystallized and a small liquid residue was rejected. The solid material was again allowed to melt gradually and the melt was removed in small successive portions, in order to wash the crystals remaining as thoroughly as possible, until one-half had been removed. The bromine remaining in the solid condition, which now represented one fourth of the original quantity, melted at −7.2°C. This was designated as D. C. C. and was the only sample that entered into the final work. The liquid quarter which was treated further in a manner similar to the rest was used in the preliminary determinations. It is designated as D. C. L. for convenience.

The bromine was next converted into hydrobromic acid by means of hydrogen. The hydrogen was generated electrolytically from a solution of pure potassium hydroxide, using four twenty-ampere cells in parallel. From the hydrogen generator the gas bubbled through the heated bromine. The mixture of bromine and hydrogen then passed through a heated glass tube about one meter in length which was filled with platinized quartz (prepared with bromplatinic acid). The hydrobromic acid formed was absorbed by conductivity water. With the proper adjustment of the rate of the hydrogen evolution and the temperature of the bromine, the mixed gases burned as a continuous flame at the entrance to the platinized quartz tube, and neither constituent remained in excess at the end of the system, which was sealed by a water trap. While it was possible to obtain the hydrobromic acid practically colorless it was considered best to carry the reaction on so that a slight excess of bromine remained with the acid.^8^ The apparatus was of glass throughout, all connections being made by ground glass joints. The hydrobromic acid thus formed was distilled twice. In the first distillation the excess of water and the free bromine were removed. No iodine could be detected in the advance portion of the distillate. The portion coming over colorless and of the constant boiling concentration was collected. This was then redistilled with some bromplatinic acid^9^ made from the same material.

**Fig. 1:**
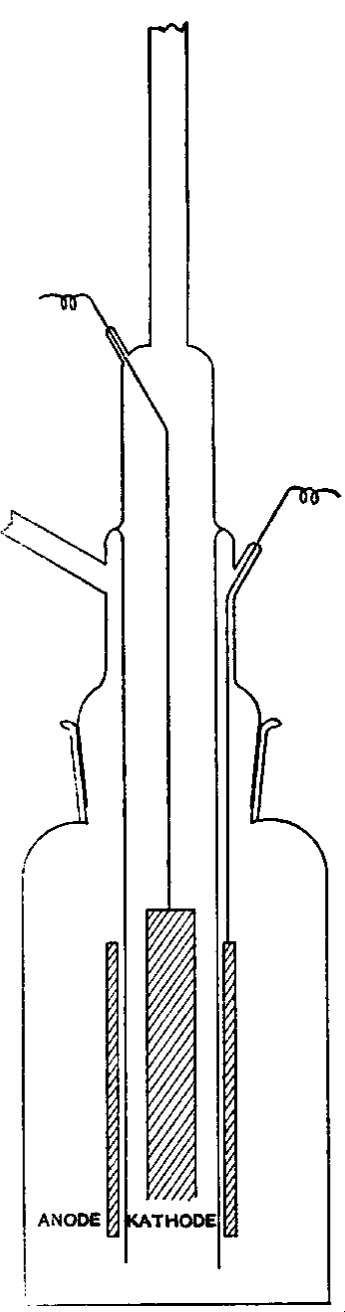


The hydrobromic acid was then decomposed into hydrogen and bromine by electrolysis. The apparatus is shown in Fig. 1.

The electrodes were of Acheson graphite, the lead wires of silver, and otherwise nothing but glass entered into the composition of the apparatus. When nearly all of the hydrobromic acid had been decomposed the bromine was distilled from the solution. No chlorine could be detected in any of the residues from the distillation.

The bromine so obtained was once more distilled from a solution of potassium bromide made from the same material. One-fourth of this bromine was again converted into hydrobromic acid and then redistilled several times with the addition of water and some bromine, and then with the addition of bromplatinic acid. The first portion of the distillate, containing dilute acid and free bromine, was rejected in every case. The acid was then kept in well seasoned bottles which had been used for the same materials for a long time previously. The remainder of the bromine which was not converted into hydrobromic acid was distilled twice from a solution of HBr and once from bromplatinic acid. The material was then set aside. Directly before use both bromine and hydrobromic acid were distilled at least once in every instance.

The following scheme will show at a glance the processes which the material underwent during purification :

**Figure:**
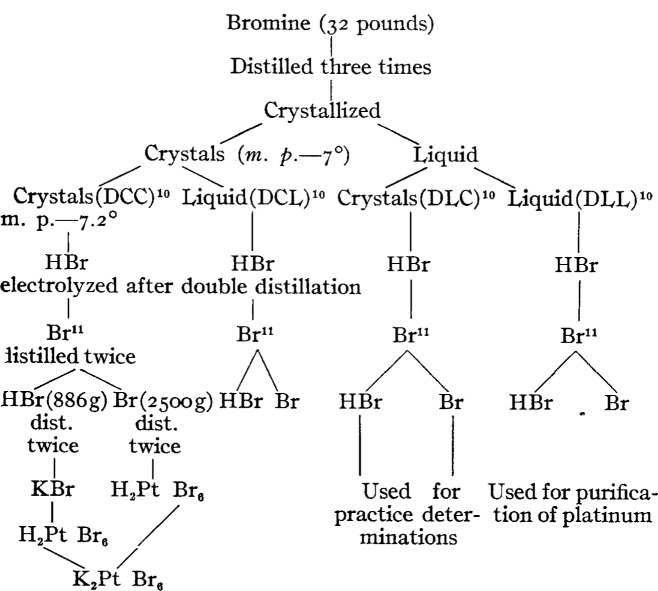

### Potassium bromide

The potassium for the potassium bromide was obtained from the oxalate. The original plan was to prepare the hydroxide by electrolysis of potassium oxalate, using a mercury cathode and a platinum anode. It was necessary to obtain considerable quantities (kilograms) of the hydroxide, and rather large current densities were therefore employed in the trial experiments. The mercury amalgam obtained was decomposed by allowing it to remain in contact with pure water in a large platinum dish. About 500 g of potassium hydroxide were prepared in this way. Upon examination the product was found to contain, besides platinum, considerable quantities of mercury, and the scheme was abandoned.

C. P. potassium oxalate, containing a trace of chlorine only, was recrystallized with centrifugal drainage of the crystals until the ordinary tests failed to show chlorine. Recrystallization was then continued with more rigorous examination of the product. Since a preliminary test showed that silver chloride is appreciably soluble in potassium oxalate solutions, especially when these are rather concentrated, the oxalate was converted into carbonate by ignition. The carbonate was then distilled with pure sulphuric acid and the distillate was examined for chlorine by means of silver nitrate in the nephelometer. After the fifth crystallization the mother liquor contained three parts of chlorine in a million of salt. The material still contained some of the constituents from the glass. Potassium oxalate solutions seems to attack glass especially, and it is not possible to obtain a product which will dissolve to a perfectly clear solution while working in glass. It was therefore necessary finally to recrystallize several times in platinum.

The potassium bromide used in the purification of the platinum was obtained from this oxalate and purified bromine DCL and DLC. The bromide used in the final determinations was made by converting the oxalate into carbonate by ignition in an electric furnace. This was dissolved and filtered; the solution was neutralized with pure hydrobromic acid, crystallized by evaporation after having been made alkaline, and the salt was then fused in platinum.

### Formic acid

In the reduction of the impure platinum salts in the course of the purification of the platinum, commercial sodium formate was used. For the purer material purified formic acid and potassium carbonate, the latter prepared as for the bromide, were used. The commercial formic acid, which contained some chlorine, was first distilled with silver carbonate. The distillate was chlorine free. It was then fractionally distilled four times and the portions having a density 1.21 to 1.22 were collected. The acid was then recrystallized a number of times and the portion having a melting point of about 8° C was finally used.^12^

### Platinum

Part of the platinum used had been purified for the work on chlorine. About 250 g of platinum were used in all. The platinum was first converted into chlorplatinic acid by aqua regia, the greater part of the iridium being removed by reduction with caustic soda according to Schneider and Seubert.^13^ It was precipitated three times as potassium chlorplatinate, the precipitate being reduced in each case with formate and the finely divided platinum black being boiled with dilute hydrochloric acid for some time in order to remove as much of the iron as possible. Next the platinum was converted into bromplatinic acid by means of commercially pure hydrobromic acid and bromine. Some iridium remained insoluble here and was removed by filtration. After the excess bromine had been removed by distillation, potassium bromide was added and the precipitated potassium bromplatinate was collected on a porcelain filter, washed, and then reduced with pure formic acid and potassium carbonate. The finely divided platinum black was heated with hydrobromic acid^14^ for seven or eight hours to remove baser metals.

Before being dissolved again the platinum black was finally heated to about 400° C in order to render iridium less soluble and remove volatile products, such possibly as occluded carbon monoxide from the formic acid.

In all, the platinum had been converted into the chlorplatinate three times and into the bromplatinate seven times. The seventh time the bromplatinic acid was prepared by dissolving the platinum electrolytically in hydrobromic acid in an apparatus similar to the one used for the preparation of chlorplatinic acid.^15^ During the course of the determinations the platinum was reduced and converted into the bromplatinate three times more. In spite of this long course of purification the platinum was not absolutely free from other platinum metals. (This would, however, not affect the bromine value.) Each time the platinum was dissolved a residue of irridium remained. This was especially noticeable if the platinum had been strongly heated. On the other hand the mother liquor from the bromplatinate always contained platinum metals and traces of iron. It was evident that these residuals were gradually decreasing in quantity. Thus the mother liquor from the last precipitation made, in which 1200 g of bromplatinate were prepared, gave, besides platinum, a few milligrams of other metals consisting mainly of rhodium. Among other things traces of lead could always be detected.

### Potassium bromplatinate

The potassium bromplatinate was prepared by the action of bromine and hydrobromic acid on platinum and precipitation of the bromplatinic acid formed with potassium bromide.

After the platinum black had been washed, dried, and heated to about 400° C it was transferred to 250 cc glass-stoppered bottles. The same bottles had been used in the purification of the platinum and were well seasoned. Seventy to eighty grams of platinum were taken to each bottle. Bromine and hydrobromic acid were then mixed in the ratio 2HBr : 4Br. Enough of this mixture to combine with all of the platinum and about 30 per cent in excess were then added to the platinum. After waiting a moment and shaking the mixture up a few times, in order to allow occluded gases to escape, the bottles were securely stoppered. They were then placed in beakers containing distilled water and heated on the steambath. After a variable period, depending on the physical condition of the platinum,^16^ the metal was completely dissolved.

The solutions of bromplatinic acid from the various bottles were then combined and the excess of bromine was distilled off. After dilution with sufficient water to prevent crystallization the bromplatinic acid was filtered through a platinized Gooch crucible. A small quantity of undissolved material, iridium, usually remained on the filter. The solution was then cooled with ice and added drop by drop to a nearly saturated solution of potassium bromide. An excess of 35 per cent of potassium bromide was used for the precipitation of the bromplatinate. The bromplatinic acid as prepared contained a considerable excess of hydrobromic acid, the potassium bromide was used in excess, and the solutions were kept cold and as concentrated as possible in order to prevent hydrolysis. During the precipitation the material was stirred constantly and was then left standing in the cold solution several hours, with occasional stirring. When freshly precipitated the salt was usually yellow to orange, the color being lighter with the more finely divided precipitate. In course of time the precipitate darkened, usually becoming a beautiful ruby color. The material was not well adapted to centrifugal drainage. It was collected on a Büchner funnel, using S. and S. No. 575 hardened filter paper, washed first with a saturated solution of potassium bromide and then with cold distilled water. It was next dried in a vacuum and then on the steam bath. The bulk of the material was then kept in this condition.

About 200 g of this material were taken for each determination. This quantity of air-dried bromplatinate was transferred to a hard glass tube with ground-glass stoppers and heated to 500° C in an electric furnace. The tube was evacuated to a pressure of 20 mm of mercury, and pure dry air was drawn over the bromplatinate at this pressure. After at least eight hours heating at this temperature the material was transferred, with as little exposure to the air as possible, to the apparatus in which the determination was to be made. The opening through which it had been introduced was immediately sealed off. The apparatus was then evacuated to a few thousandths of a millimeter and heated to 400° C. Any gas which accumulated was removed from time to time. It was then left overnight at about 300° C. Properly prepared and pure bromplatinate would not show an appreciable increase in pressure in the apparatus by the next morning. The changes, if any, amounted only to thousandths of a millimeter. After having been prepared in this way the apparatus containing the bromplatinate was allowed to cool, was cleaned and rinsed with distilled water, and set aside to be weighed. The bromplatinate used in the last five determinations was prepared from hydrobromic acid and potassium bromide, which resulted from the preceding determinations, and from bromine DCC.^17^

### Water

The water used was three times distilled. In the third distillation only the middle portion of low conductivity was collected. The final product had a conductivity of 1 to 1.5×10^−6^. It was kept in a large, glass-stoppered bottle, which was thoroughly aged, and was protected from atmospheric impurities by a soda lime bulb and by a sulphuric acid tube.

### Balance and weights

The balance was a Ruprecht, designed to carry a kilogram in each pan. The manner of drying the air of the balance case has been described in the paper on the atomic weight of hydrogen, by Noyes.^18^

A slight change was made in the housing of the balance case.. The balance, with its glass inclosure, was placed in a large copper case, which was blackened inside and out and formed a box which was almost air tight. The whole apparatus was arranged to exclude any radiation while the weighing was being made.^19^

A small window in the case and a lens permitted reading the swings of the balance pointer. A small cardboard funnel, white on the inside and blackened on the outside, illuminated the balance scale without permitting any light to enter the rest of the balance. The metal case itself was grounded, by a wire connection, to the laboratory piping. A small beaker containing radioactive pitchblende was placed inside of the balance case. In this way unequal effects on the balance beam, due to radiations of various kinds, or to electric charges, were eliminated as much as possible.

The three pieces of apparatus, the palladium tube, the platinum tube, and the absorption or hydrobromic acid tube, were weighed against counterpoises of glass. In the case of the palladium tube, by means of which the hydrogen was weighed, the counterpoise was made from the same lot of glass and was shaped as nearly as possible of the same form, in order to duplicate the surface as well as the volume of the palladium tube. Its volume was within 0.15 cc and its weight within 3.5 g of that of the palladium tube. The only vacuum correction necessary on the weight of the hydrogen was that due to these small weights.

The counterpoises for the bromplatinate tube and the hydrobromic acid tube were within a cubic centimeter of the volume of these respective pieces of apparatus. The weights employed here were of necessity larger, since the changes in weight amounted to 80 g and the vacuum corrections were correspondingly larger. It may be noted here in passing that the allowable error on these two pieces of apparatus was eighty times that of the hydrogen tube.

Before being weighed all pieces of apparatus were rinsed with distilled water, wiped, and transferred to a glass case through which dry air was passing. Weighings of the hydrogen tube were made after the tube had been suspended in the balance overnight. With the other pieces at least two hours were allowed to elapse between handling and weighing the apparatus. The air current passing through the balance was shut off 15 minutes before a weighing was made. Weighings were made by swings, the sensibility of the balance being determined for each weighing, the readings being taken over a range of 2 mg. The weight taken was the average of four to five observations of about 10 swings each. For the hydrogen, observations were made at separated intervals of time. These individual observations rarely differed by as much as 0.05 mg and usually only by 0.01 or 0.02 mg. With every weighing of the hydrogen tube the zero point of the balance was determined.

The balance and the method of weighing have been described in some detail here for the reason that it seems that the accuracy of the determinations is limited chiefly by the accuracy with which the hydrogen may be weighed. On account of the large ratio small errors on the other constituents practically vanish.

### Weights

The weights used were those which served in the work on chlorine. They were twice calibrated during that investigation and a third time during the investigation on bromine. There was no appreciable change in the corrections.

## METHOD OF CARRYING OUT THE DETERMINATIONS

The method of carrying out the determinations and the apparatus used was essentially the same as that used in the chlorine work.^20^

The palladium tube used was capable of absorbing about 2.3 g of hydrogen. The absorption tube for the hydrobromic acid was somewhat larger than in the previous investigation, each bulb having a capacity of about 135 cc. While the theory of the determination was precisely the same as that of the previous work the actual manipulation was considerably more difficult. The first 13 determinations which were made gave results having no value whatever, as far as accuracy is concerned. In some cases the reaction would not proceed with reasonable speed; in others it was not possible to reduce the pressure in the system at the end of a determination. In the latter case it was not possible to find and determine the hydrogen left over, since the hydrobromic acid also present immediately fouled the mercury pump. Another very serious error lay in the accidental diffusion backward of hydrobromic acid to the palladium tube. This acid could be found after the determination had. been carried to an end by passing hydrogen over the heated palladium. The hydrobromic acid was given off from the palladium much more slowly and imperfectly than hydrochloric acid under the same conditions.^21^

Considerable time and labor were spent in overcoming these difficulties. The backward diffusion of hydrobromic acid was finally prevented by the use of anhydrous copper sulphate as described further on. The condensation of the hydrobromic acid and its transfer to the water was effected in two operations instead of one. This made it possible to bring the apparatus to a satisfactorily high vacuum at the close of the determination. With these changes in manipulation the large discrepancies between the weight of H + Br and the weight of HBr, which has been usual before, disappeared. On account of these and other apparent errors all determinations made previous to these changes have been disregarded as a whole.

**Fig. 2:**
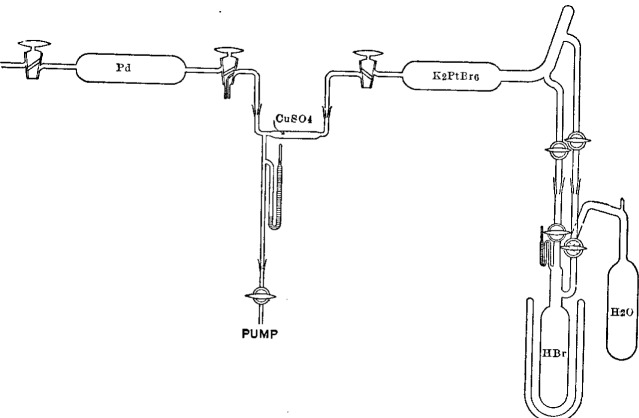


The sketch of the apparatus, Fig. 2, shows the relative arrangement of the parts for a determination. The connection between the hydrogen and the bromplatinate, which at the same time formed the connection to the vacuum pump, contained a section about 5 cm in length which was filled with anhydrous copper sulphate.^22^

After the different parts of the apparatus had been sealed together by means of a hard cement the connections were evacuated to a few thousandths of a millimeter residual pressure. The system was then allowed to stand for 20 minutes, the platinum being heated meanwhile. If no leaks showed the stopcock to the pump was closed and the actual transfer of material was commenced. The condensation bulb for the hydrobromic acid was surrounded by liquid air, and the hydrogen was allowed to enter the bromplatinate tube gradually. The flow of the hydrogen was regulated by the temperature to which the palladium tube was heated and by the adjustment of the stopcocks. The bromplatinate was kept at 250°C during the main part of the determination and raised to 400°C toward the end. The white, solid hydrobromic acid appeared in the condensation bulb immediately after the hydrogen was admitted to the bromplatinate. After a while the bulb was filled with a white snowy mass and the condensation became slower, the pressure rising at the same time. From this period onward the process had to be interrupted from time to time in order to allow the hydrobromic acid to melt partially and sinter together into a more compact mass. As the pressure in the apparatus rose considerably during this manipulation it was necessary to close the stopcock between the bromplatinate and the copper sulphate during the process.

When a sufficient amount of hydrobromic acid had been formed, the stopcocks between the platinum and hydrogen tubes were again closed, and the hydrobromic acid formed was condensed as completely as possible. The small amount of hydrogen left in the connecting tube was allowed to enter the platinum tube from time to time as the pressure fell. It usually took two to three hours to reduce the pressure in the apparatus to a small fraction of a millimeter, and the process had to be hastened by alternate warming and chilling of the acid until it had sintered together to a solid block. When the pressure was very low, as was shown by a small manometer sealed on the condensation bulb, the stop cocks between the platinum tube and the hydrobromic acid tube were closed. The liquid air was lowered away from the hydrobromic acid and the pressure was allowed to rise slightly so as to give a constant stream of hydrobromic acid when the connection to the bulb containing the chilled water was opened.^23^

The hydrobromic acid passed over to the water without liquefying, except occasionally at the very end of the transfer. In three hours the 80 g were usually absorbed completely. The platinum tube had meanwhile been kept hot. After a few moments the condensation bulb was again surrounded by liquid air and the stopcocks connecting with the platinum tube were opened. The small amount of hydrobromic acid remaining was now very perfectly condensed in the empty bulb. Any small quantity of hydrogen remaining in the copper sulphate connection was allowed to enter the platinum tube and be converted into hydrobromic acid. Any hydrogen that might have been carried over to the water bulb was allowed to escape by opening the stopcock to this part of the apparatus momentarily from time to time. Any gas coming from here had to pass through the bulb cooled by liquid air, so that water vapor (the liquid was now at −15°C) could not be carried to the platinum. The small amount of hydrobromic acid condensed by this second operation was then transferred to the water.

After the determination had been brought to a satisfactory conclusion the apparatus was pumped out by means of the Sprengel pump, as far as the hydrobromic condensation bulb. This latter was examined after it had been weighed. Small traces of hydrobromic acid which had not been condensed did not get into the pump, as they were retained by the copper sulphate. Very often no gases were found on pumping. If there were any they were collected and analyzed, the gas analysis being carried on under reduced pressures. The parts of the apparatus were then allowed to come to normal temperature, taken apart, the cement was carefully cleaned from the connections, the apparatus was rinsed with distilled water, wiped and set aside to weigh. The actual determination required from 12 to 16 hours from beginning to end.

## ERRORS AND CORRECTIONS

### Hydrogen

What has been said in the previous paper^24^ with regard to the purity of the hydrogen applies to this case. The transfer of hydrobromic acid back to the palladium tube has been prevented by the use of anhydrous copper sulphate. In the ten determinations there was one exception to this. In the tenth determination 1.78 mg of HBr were obtained from the palladium.

Hydrogen was pumped from the apparatus at the end of the first and ninth determinations. The quantities were 0.05 mg and 0.96 mg respectively.

### Bromine

As far as can be seen, the bromine was as pure as could be desired and the bromplatinate was free from volatile impurities. Other halogens were looked for in every step of the process of purification. Volatile constituents were shown to be absent in the bromplatinate as in the case with the chlorplatinate.^25^

An occasional correction was necessary for bromplatinate carried over mechanically to the condensation bulb at the very beginning of the determination. This remained here at the end of the experiment and was removed and weighed after weighing the apparatus. This correction was applied in determinations 6, 8, and 10 and amounted to 155.1, 43.6, and 64.8 mg, respectively.

### Hydrobromic Acid

At the temperatures and under the conditions used there was no evidence whatever of dissociation of the hydrobromic acid, and no free bromine was carried over. The weight of the hydrobromic acid was corrected for the weight of the bromplatinate transferred mechanically, where this was necessary, as is described for the corrections on bromine. A further correction was necessary for the amount of acid absorbed by the copper sulphate. Some hydrobromine acid was found here at the end of every determination. Its amount was determined by titration with N/10th silver nitrate solution. The quantities found were respectively 59.95, 3.30, 8.40, 14.17, 32.28, 4.73. 3.09, 9.32, 6.48, and 61.56 mg.

### Agreement Between Weights

The agreement between the sum of the weights of hydrogen and of the bromine and the weight of the hydrobromic acid served as a final check on the accuracy of manipulation. In the 10 determinations the maximum difference was 2.5 mg. The average difference was 0.26 mg. The sum of the differences for 10 determinations was −2.63 mg. This represents one part in 275 000 on the hydrobromic acid.

## RESULTS

The results obtained are given in Table I. The column marked discrepancy gives the differences between H + Br and HBr. The remainder of the table is self-explanatory.

In the 10 experiments 9.00369 g of hydrogen were combined with 714.05722 g of bromine and yielded 723.05828 g of hydrobromic acid. The value obtained from these two sums is respectively 79.307 (1) and 80.306 (9).

The final ratio obtained from these figures for H : Br is 79.306 (7), with a probable error of 0.0022. The ratio found from H : HBr is 80.306 (4), with a probable error of 0.0018. Combining the two the value is 79.306 (6) ± 0.0014. The numerical value of the probable error is somewhat larger than that of the chlorine ratio obtained in a similar manner. Relatively it is approximately the same or even somewhat smaller.

In connection with this determination of the bromine : hydrogen ratio, it may be of interest to note the relation of this value to the value determined for chlorine previously.

Richards and Müller^26^ obtained a ratio between bromine and chlorine equal to 0.44369, while Baxter^27^ obtained 0.44367, for the same ratio. Combining the average of these two ratios, 0.44368 with the value here obtained for bromine we get 79.3066 × 0.44368=35.186(7) for the hydrogen : chlorine ratio. The value obtained by Noyes and Weber was 35,184. Dixon and Edgar obtained 35,195^28^ and Kdgar in the second series obtained 35.193^29^

Taking the atomic weight of hydrogen^30^ as 1.00779, the value for bromine on the oxygen basis becomes 79.924, as against the value 79.920 given by the International Commission for 1912.

Washington, November 1, 1912.

## Figures and Tables

**TABLE I:** 

Experiment	Grams H	Grams Br	Grams HBr	Discrepancy in mg	H: Br	H:HBr
						
1	0.77300	61.28837	62.06052	−0.85	79.2863	80.2853
2	0.86060	68.25033	69.11144	+0.54	79.3055	80.3061
3	0.77607	61.54733	62.32198	−1.42	70.3064	80.3046
4	0.96927	76.88221	77.85135	−0.15	79.3197	80.3195
5	1.07545	85.29562	86.37092	−0.15	79.3114	80.3114
6	0.99689	79.06834	80.06424	−0.99	79.3150	80.3140
7	0.74966	59.45275	60.20500	+2.59	79.3063	80.3097
8	0.98161	77.85554	78.83758	+0.43	79.3141	80.3145
9	1.00131	79.39533	80.39658	−0.06	79.2915	80.2914
10	0.81983	65.02140	65.83867	−2.56	79.3108	80.3077
						
	9.00369	714.05722	723.05828	−2.63	79.3067	80.3064
					±0.0022	±0.0018
					**Combined**	
					79.3066	±.0014

## Footnotes

1 This Bulletin, 4, p. 345; 1908. (Reprint No. 81); J. Am. Chem. Sec. 30, p. 13; 1908.

2 Constants of Nature, Part 5, Smithsonian Publication 1923; 1910.

3 Abegg, Handbuch der Anorganischen Chemie., vol. 2, 1; 1905.

4 J. Am. Chem. Soc., 33, p. 35; 1911.

5 This Bulletin 4, p, 179; 1908 (Reprint No. 77.). Noyes, J. Am. Chem. Soc., 29, p. 1721.

6 It was first attempted to remove any chlorine present by shaking the rather concentrated solution of barium hydroxide with silver oxide. Considerable quantities of silver went into solution, however, and this method was abandoned. It may be well to call attention to the advantages of the barium hydroxide electrolyte at this point. Aside from the fact that it is usually of greater purity than sodium or potassium hydroxides, it offers certain advantages in its manipulation. A solution saturated at about 50*°* C. was used. On account of the great Variation of solubility with the temperature, this deposits a considerable quantity of crystals in the electrolytic cell upon cooling. As the current is sent through the apparatus the solution warms up slightly, some of the crystals redissolve and the resistance goes down. Within certain limits the resistance adjusts itself to the load, Thus in the apparatus used the initial resistance, including a certain outside resistance, was 40 ohms. After the barium hydroxide had dissolved completely the resistance under the same conditions was 10 ohms. The apparatus was connected to a 120-volt lighting circuit and 12 amperes could be sent through the apparatus, giving 0.448 g of hydrogen per hour. The apparatus was kept well cooled by a stream of water when running at this load.

7 The Mallinckrodt Chemical Co. consented to prepare this especially for the purpose. It consisted of about 40 pounds which represented the middle fraction of a large quantity of their purest bromine. The analysis made at the factory according to the method of Andrews showed at the outset a quantity of chlorine which was negligible. Iodine was present in very small quantity. Besides this the chief impurity was a trace of organic material.

8 Some platinum was always found with the acid formed. Whether this was carried over mechanically by the heavy vapor of hydrobromic acid is not certain. The arrangement of the apparatus did not make this seem likely. Wöhler and Spengel, Zs. Anal. Chem., 50 p. 167: 1911, have shown that platinum is volatile when heated in a current of chlorine, less so when heated in a current of bromine.

9 The original bromine contained organic matter. This concentrated largely in the portion which liquefied first and which was rejected. There was not enough of this material obtained to examine it very thoroughly but a substance was found among other things, which gave a bromplatinate. The bromplatinic acid was added to retain this impurity. That aliphatic bromine compounds and cyanogen are at times present in bromine is known.

12 As is the case with acetic acid, the melting point of formic acid changes much more markedly than the density after the acid has reached a concentration of about 95 per cent. Taking the molecular lowering of the melting point of formic acid as 29° C. 5 per cent of water would lower the melting point about 7.5° C. The further purification was therefore followed by means of the melting point.

13 Graham-Otto’s Lehrbuch II, 4, p. 1153; 1889.

14 The finely divided platinum black always dissolved to a noticeable extent in the hydrobromic acid. This treatment is not particularly well adapted to the removal of baser soluble metals, such as iron. Reprecipitation of the platinum as the complex salt is much more effective. In general the extreme tenacity with which platinum black retains other substances was one of the factors which had to be taken into consideration. With quantities of over 200 g at least 24 hours of continuous washing with hot water was necessary before the wash water wasfreefrom bromine. Platinum black thus treated would, nevertheless, give off large Quantities of bromine vapors when heated to 300°–400° C. The long-continued washing was necessary to remove formic acid and potassium bromide, which would interfere subsequently in the preparation of the bromplatinic acid.

15 Weber. Preparation of chlorplatinic acid. This Bulletin, 4, p. 365: 1908 (reprint No. 82). J, Am, Chem. Soc., 30, p. 39.

16 If the platinum was very finely divided and had not been heated much above 300° C an 80 g lot of the metal would be dissolved completely after 24 hours heating on the steam-bath. At 500° C or so the platinum black rapidly sinters and becomes gray. The same quantity of this gray sponge requires a week to ten days continuous heating before it is dissolved completely. This marked difference in behavior is worth noting in connection with the attempted separation of the platinum metals by their difference in solubility in various acids or mixtures of acids. At any early stage of purification of the platinum an accident caused considerable loss of time and of some material. A greater quantity of platinum than usual was taken. For this reason the mixture of bromiue, hydrobromic acid and platinum was placed in cracked ice after standing open for a few moments. The bottle was then stoppered. Shortly afterward the material in the bottle commenced to seethe and before anything could be done the bottle burst, throwing the material about with great violence. The platinum used had been prepared some time before and had been set aside in a desiccator. When taken for use it had been moist in this instance. The moisture determination previously made showed 0.65 per cent water. Occluded gases had been given an opportunity to escape while the platinum stood open in the bottle with the bromine mixture. There were no indications of such behavior at any time previous to or subsequent to this single instance. The pressure in the bottles never rose above that due to the vapor tension of the bromine and hydrobromic acid mixture at temperatures near 100° C. Possibly the platinum in this case was in a condition similar to that investigated by Cohen and Strengers, “ Explosive Platinum Metals,” Zs. f. Phys. Chem., 61, p. 698.

17 During the purification of the materials, some trial runs were made with bromplatinate prepared from the bromine which has been designated as DLL. This material gave off vapors when heated to 500° C and was in general more unstable than the salt prepared from purer materials. Neither could so good a vacuum be obtained with this salt. On attempting to carry out a determination, a permanent gas was obtained which made it impossible to carry the determination to a satisfactory conclusion. The actual weight of this gas was small and amounted to but a centimeter pressure in a volume of 150 cc, not enough to affect the results. Still it acted as a gas plug and effectually blocked the condensation of the hydrobromic acid formed. Subsequent examination of the platinimi salt showed the presence of a small amount of organic matter, which could not be identified with certainty. Organic matter had also been found in the bromine DLL, and none of this material was used in the final determinations.

18 J. Amer. Chem. Soc., 29, p. 1723. This Bulletin, 4, p. 179; 1908. (Reprint No. 77)

19 Manley, Trans. of the Royal Society, 210, p. 387, has shown that small temperature fluctuations of the balance beam, due to radiation, affect the zero of the balance.

20 Loc. cit.

21 Loc. cit.

22 The copper sulphate was prepared by recrystallizing the commercial salt until free from halogen. It was then dehydrated, first at low heat, finally adding some sulphuric acid and heating to about 350°C. This material is not affected by hydrogen at normal temperatures and absorbs hydrobromic acid greedily. The salt turns almost black where the hydrobromic acid strikes it. At very low pressures the hydrobromic acid may be liberated by gentle warming, leaving the copper sulphate unaffected. The salt therefore served admirably as an indicator for hydrobromic acid and as a trap to prevent its reaching the palladium tube.

23 After some hydrobromic acid bad condensed in the water it was cooled by a freezing mixture instead of by ice alone. For this purpose ice and alcohol were used. This is much more agreeable to work with than either salt or sulphuric acid and ice. One may quite readily attain −15°C and even lower.

24 Loc. cit.

26 Richards. Atomgenichte p. 825 (1909).

27 Loc. cit.

28 Phil. Trans. 205A. p. 169 (1909).

29 Proc. Roy. Soc., 81A. p. 216 (1908).

30 Clarke: Recalculation of Atomic Weights, p. 41, 1910.

